# Tissue specific RISC-loading reassesses small RNA functionality in developing pepper fruit

**DOI:** 10.1007/s11103-026-01742-6

**Published:** 2026-07-11

**Authors:** Ágnes Dalmadi, Péter Gyula, Auwalu Abdu, András Kis, Fabio Miloro, Jeannette Bálint, György Szittya, Zoltán Havelda

**Affiliations:** 1 Department of Plant Biotechnology, Hungarian University of Agriculture and Life Sciences, Gödöllő, Hungary; 2 Institute of Plant Biology, HUN-REN Biological Research Centre, Szeged, Hungary; 3 Doctoral School of Natural Sciences, Biological Science Program Gödöllő, Hungarian University of Agriculture and Life Sciences, Gödöllő, Hungary

**Keywords:** Small regulatory RNAs, ARGONAUTE loading, Pepper fruit, Development

## Abstract

Small regulatory RNAs (sRNAs), including small interfering (si) and micro (mi) RNAs, are components of the RNA interference (RNAi) pathways regulating vital processes. These various sRNAs act via specialized executor ARGONAUTE (AGO) proteins constituting RNA induced silencing complexes (RISCs). In *Arabidopsis*, miRNAs load mainly into AGO1, while 24-nucleotide (nt) long siRNAs load into AGO4, typically. However, a large subset of sRNAs is present in the cytoplasm in AGO-unbound form. To uncover the potential importance of tissue specific AGO-loading efficiency of sRNAs in an economically important plant we used pepper fruits, dissected into pericarp, placenta, and seed and the ratio between AGO-loaded and -unloaded sRNA pools was investigated with high throughput sequencing. In agreement with the previous studies, variable loading capacity of sRNAs was discovered with an abundant AGO-unbound pool. The investigated pools showed a surprisingly high prevalence of 21-22-24-nt long siRNAs even in the high molecular weight RISC. The dynamically expanding pericarp exhibited extremely high 21-nt long miRNA content. Moreover, comparing the different tissues, a drastic redistribution or elimination of 21- and 24-nt sRNAs in various sRNA-protein complexes and changes in unbound sRNA pools were observed demonstrating the highly dynamic alteration in AGO-loading properties of the sRNAs. These data imply that during fruit development sRNAs are dynamically sorted into various executor complexes in a controlled manner. This finding also implies that analysis of the content of AGO-sRNA complexes together with the AGO-unbound population of these sRNAs can be a useful tool to identify potentially functional sRNAs.

**Supplementary Information:**

The online version contains supplementary material available at 10.1007/s11103-026-01742-6.

## Key message

Size-separation analyses of seed, placenta and pericarp extracts of developing pepper fruit reveal altered presence and redistribution of small regulatory RNAs in various executor complexes indicating a dynamic RNAi mediated control during the developmental process. Sequence analyses of small regulatory RNAs associated with executor complexes can open a new approach to identify potentially biologically relevant, active small RNA species.

**Supplementary Information:**

The online version contains supplementary material available at 10.1007/s11103-026-01742-6.

## Introduction

RNA interference (RNAi) is a widespread regulatory mechanism. In plants, various RNAi pathways play indispensable roles in diverse developmental processes and in responses to biotic and abiotic stress factors (Vaucheret and Voinnet 2024; Yu et al. 2026). These small (s)RNA mediated processes involve different types of small RNA species and specialized executor proteins, which act in a sophisticated molecular network often showing redundancies in their functions (Zhan and Meyers 2023).

Micro (mi)RNAs originate from RNA Polymerase II (Pol II) transcribed precursor RNA molecules (pri-miRNAs) having a specific hairpin-like secondary structure. Pri-miRNAs are capped and polyadenylated, then processed at the hairpin structure typically by DICER LIKE 1 (DCL1) to generate pre-miRNA intermediates, and subsequently 20-24-nucleotide (nt) long double stranded miRNA/miRNA* duplexes. The main executor molecules of RNAi are the ARGONAUTE (AGO) proteins, which are specialized in diverse pathways. The mature miRNA strand of miRNA/miRNA* duplexes is typically loaded into AGO1 protein containing RNA induced silencing complexes (RISCs), while the miRNA* strand is eliminated. The miRNA loaded RISCs are directed to the target mRNA molecules in a sequence-specific manner, triggering their negative regulation via cleavage or translational inhibition (Rogers and Chen 2013).

In contrast to miRNAs, 24 nt long small interfering (si)RNAs are generated by the action of DCL3. Plant specific Pol IV is responsible for the production of single-stranded RNAs that will serve as precursors of DCL3 generated siRNAs. The activity of Pol IV is tightly coupled to RDR2 (RNA-DEPENDENT RNA POLYMERASE 2), which is capable to transcribe the newly synthesized Pol IV transcripts to double-stranded RNAs (dsRNAs). The generated 24-nt siRNAs are predominantly loaded into the executor complex to play fundamental roles in maintaining genome integrity by regulating the *de novo* DNA methylation (RNA-directed DNA methylation (RdDM) of transposable and repetitive elements. The main executor proteins associated with this pathway are AGO proteins belonging to the AGO4 clade (AGO4, AGO6). Phased (pha)siRNAs, however, are derived from Pol II-dependent mRNAs which are cleaved by a special class of miRNAs/siRNAs. This cleavage renders one of the cleavage products to be a substrate of RDR6/SUPPRESSOR OF GENE SILENCING 3 (SGS3). The resulting dsRNA is then cleaved by DCL4 endonuclease following a distinctive, regular (phased) pattern starting from the cleavage site. A subset of phasiRNAs, the trans-acting siRNAs (tasiRNAs) act mainly at the post-transcriptional level by negatively regulating their target transcripts including gene families which encode disease resistance proteins or transcription factors (Shivaprasad et al. 2012).

Size-separation of crude extracts of *Arabidopsis* flower and leaf sample revealed that 21-nt miRNAs mainly associated with a high molecular weight (HMW) RISC complex containing AGO1 (Dalmadi et al. 2019). The 24-nt long siRNAs were present typically at low molecule weight (LMW) RISCs containing AGO4. Intriguingly, we also identified a large number of abundant, intact AGO-unbound sRNAs ranging from 21- to 24-nt in size. This finding revealed a controlled post-production regulatory mechanism determining the loading efficiency of particular sRNAs into various AGO-RISCs. It was shown that structural feature of pre-miRNA stem-loop structures, especially the structures of the various miRNA/miRNA* duplexes are the major factors determining the efficiency of AGO-RISC loading of a particular miRNA (Dalmadi et al. 2021, 2025). This control mechanism can determine the biologically active portion of the produced miRNAs in the given cellular environment since miRNAs sorted into the AGO-unbound pool (free) cannot be considered as biologically active sRNAs. The regulatory action sorting 24-nt siRNAs to LMW-RISC or AGO-unbound pool is not known. To date, only *Arabidopsis* data were available describing the distribution of sRNAs in HMW-, LMW-RISCs and protein unbound pools.

The fruit, this highly specialized complex organ composed of various tissue types requires sophisticated, multi-layered developmental processes during its formation. Understanding the fine molecular mechanisms of pepper fruit development is important from both practical and scientific points of view since pepper fruits provide essential nutrients and minerals for a healthy and balanced diet. Pepper fruit related regulatory sRNAs were previously investigated at genomic level using total RNA extracts (Liu et al. 2017; Taller et al. 2018; Lopez-Ortiz et al. 2021). Here we provide high resolution tissue specific data identifying various RISC-related and protein unbound sRNA pools in developing pepper fruits. We show that the regulated distribution of sRNAs amongst HMW-, LMW-RISC related and unbound sRNA pools also characteristic to developing pepper fruit and exhibits tissue specific variances. Moreover, we demonstrate that this experimental approach can help to identify potentially active sRNA species, to highlight the importance of yet uncharacterized sRNAs, and to elucidate the functionality of known miRNAs.

## Materials and methods

### Gel-filtration assay

Fast protein liquid chromatography (FPLC) based size separation of molecular complexes using Superdex-200 column was performed as described previously (Lakatos et al. 2004; Varallyay et al. 2010) with minor modifications. Crude extracts were prepared from 0.6 g of placenta and pericarp samples or 15 seeds originated from 28 days old pepper fruits (*Capsicum annuum* ‘Fehérözön’). Input samples were prepared from the crude extract prior to injection to the column. Collection of the fractions began 23 min following the injection (equivalent to 20 fractions). To the High-Throughput Sequencing (HTS), fractions between 6 and 11, 20–24 and 30–36 as High Molecular Weight RISC (HMW-RISC), Low Molecular Weight RISC (LMW-RISC) and unbound pools were combined and used to produce RNA samples, respectively. RNA was extracted with phenol-chloroform method from all samples (Dalmadi et al. 2019).

## RNA extraction and protein sample preparation

For small RNA analyses, 0.1–0.2 g of the indicated plant material was collected, homogenized in an ice-cold mortar in 650 µl of extraction buffer (0.1 M glycine-NaOH, pH 9.0, 100 mM NaCl, 10 mM EDTA, 2% sodium dodecyl sulfate, and 1% sodium lauroylsarcosine) and was used for RNA extraction with the standard phenol–chloroform method. Protein samples were produced from 0.2 g plant tissues homogenized in 500 µl of extraction buffer and supplemented with an equal amount of 2X Laemmli buffer. Samples were then centrifuged for 5 min following the boiling for 5 min. Gel-filtration samples were supplemented with 1.3 ml ice cold acetone to precipitate proteins, which were recovered with centrifugation (40 min, 4 °C, 15 000 RPM), dissolved in 10 µl of Laemmli buffer and denatured with boiling.

## sRNA detection and quantification

For small RNA northern blot analyses, 4 µg of total RNA or samples of gel-filtration were separated on denaturing 12% polyacrylamide gels containing 8 M urea and transferred to Hybond NX membrane (GE Healthcare) with semi-dry blotting (Bio-Rad). Membranes were chemically cross-linked (Pall and Hamilton, 2008) and probed either with Locked Nucleic Acid containing (sRNA_FM_1 and can-miR-n026 on Fig. 3; sRNA_DA_1 and can-miR-n018 on Suppl. Figure 4) or DNA (sRNA_DA_1 and can-miR-n018 on Fig. 3) probes biotinylated at 5’ end. Signal was detected using the Chemiluminescent Nucleic Acid Detection Module (ThermoFisher, Cat. Kit: 89880) and images were acquired with ChemiDoc equipment in Chemi High Sensitivity mode with signal accumulation.

## Western blotting

20 µl of the *Capsicum annuum*, *Nicotiana benthamiana* and *Arabidopsis thaliana* samples or protein content of gel-filtration fractions were separated on 10% sodium dodecylsulphate–polyacrylamide gel, blotted overnight to PVDF Transfer Membrane (Bio-Rad Laboratories, 1620264) using wet tank transfer and subjected to western blot analysis. Membranes were blocked using 5% non-fat dry milk in phosphate-buffered saline (PBS) containing 0.05% Tween 20 (PBST) for 60 min. Blots were cut into two, and respective parts were incubated with anti-Histone H3 (Agrisera, AS10 710) for 1 h or with anti-AGO1 (Agrisera, AS09 527) for 2.5 h at a dilution of 1:7 500 in 1% non-fat dried milk in 1X PBST. After washing in PBST, blot was incubated with secondary goat anti-rabbit IgG HRP conjugated antibody (Agrisera, AS09 602) for 1 h at a dilution of 1:10 000 in 1X PBST with agitation. Blots were developed with High Clarity Western ECL (Bio-Rad), exposure was made using ChemiDoc (Bio-Rad) equipment in signal accumulation mode.

## High-throughput sequencing (HTS) and data analysis

To create cDNA libraries for sequencing, high quality RNA samples were purified with the phenol-chloroform method from the indicated pooled FPLC fractions. 20 µg of the samples were loaded onto separate poly-acrylamide gels, the small RNA fraction was isolated and libraries were prepared only from this fraction using the TruSeq Small RNA Library Preparation Kit (Illumina, San Diego, CA, USA) and the modified protocol described earlier (Czotter et al. 2018). Sequencing was carried out on Illumina HiSeq 2000 platform with a 50 bp, single-end chemistry by UD-GenoMed Ltd. (Debrecen, Hungary).

### Bioinformatic analysis

Raw reads were processed using cutadapt v1.9.1 with the following parameters: -a TGGAATTCTCGGGTGCCAAGG -m 20 -M 25 -q 20 --max-*n* = 0 --discard-untrimmed. The processed reads were subsequently filtered to remove tRNA and rRNA sequences using bowtie v1.3.1 (Langmead and Salzberg 2012) with parameters -l 20 -k 1 -n 2 against Rfam tRNA and rRNA sequences as a reference, while the unmatched sequences were collected by specifying the parameter –un. Nonredundant sequences were then collected and quantified for each library, followed by normalizing to Read Per Million (RPM) based on the library size. Sequences were sorted by their mean abundance across the samples, and only those exceeding 5 RPM were retained for further analysis. The remaining sequences were annotated against the previously identified pepper mature miRNA sequences (Taller et al. 2018), the Zunla RNA and TE sequences, along with the miRBase pri-miRNA sequences using patman (Prufer et al. 2008), allowing for one mismatch.

Principal component analysis of the sRNA-seq data was performed using the prcomp R package. Normalized abundances (RPM) were scaled and centered before the analysis. The plots were generated with the autoplot function of the ggfortify R package.

To visualize the association of sRNAs with the different FPLC pools (HMW-RISC, LMW-RISC and unbound), heatmaps were generated based on Z-scores. The Z-score for each sRNA abundance value was calculated to normalize distribution within each tissue, allowing for the direct comparison of sRNAs with largely different mean expression levels. The calculation of Z-scores included three steps: first, the mean abundance of the two biological replicates was calculated for every sRNA, then the mean and standard deviation of the obtained abundances across samples were calculated. Finally, from every individual value the mean was subtracted and the resulting value was divided by the standard deviation. This standardization was performed independently for each tissue to highlight the sRNA distribution across the fractions within a tissue. The heatmaps were generated with the geom_tile function of the ggplot2 R package.

## Results

### Gel-filtration experiments reveal tissue specific AGO-loading efficiency of sRNAs in developing pepper fruit

**Fig. 1:**
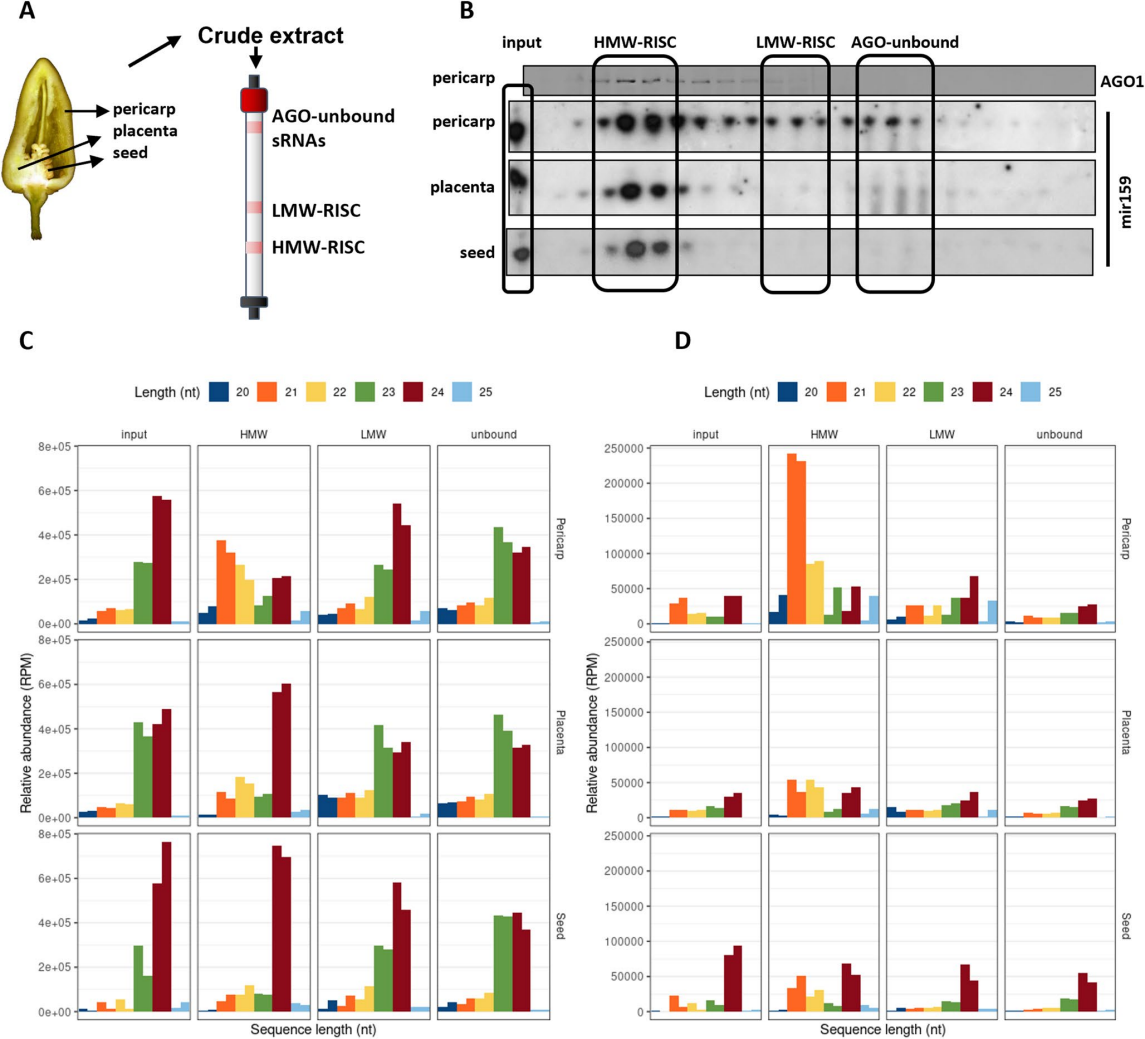
**A** Experimental setup of the gel-filtration. **B** Distribution of AGO1 protein and miR159 amongst gel-filtration fractions of 28 DPA pepper fruit tissues. Black frames indicate the input samples and the fractions corresponding to the HMW-RISC, LMW-RISC and AGO-unbound pools subjected to HTS. **C**, **D** Size distribution of all HTS reads (redundant), and of those sequences having a mean abundance higher than 5 RPM. Replica experiments are displayed side by side

To reveal tissue specific AGO-bound and unbound (free) sRNA pools and their size distribution pattern in developing 28-days-old pepper fruits, they were dissected into pericarp, placenta, and seed and the isolated tissue samples were collected and processed to crude extracts. The crude extracts were subjected to Fast Protein Liquid Chromatography (FPLC) using gel-filtration column to collect fractions representing the high-molecular-weight (HMW), low-molecular-weight (LMW) AGO-bound and unbound sRNAs, as described previously (Fig. 1A) (Dalmadi et al. 2019). The chromatograms of selected gel-filtration experiments indicate that the size separation capacity of the column was similar in all cases, as the distance between the first and the last peak is the same in all cases, and the first peak emerged in the same timepoint after injection (Suppl. Figure 1). Based on our previous experiments we defined the three pools of the fractions corresponding to HMW-RISC, LMW-RISC and the AGO-free unbound sRNAs (Fig. 1B). However, minor shifts in the fraction collections can occur. To overcome this, we collected the samples from the fraction ranges exhibiting the highest specific contents of sRNAs characteristic to the HMW, LMW and unbound sRNA pools. The position of HMW-RISC was further supported by AGO1 specific western blot of pericarp gel-filtration samples (Fig. 1B). Firstly, the collected fractions were analyzed for their miR159 content. In agreement with the previous results (Dalmadi et al. 2019), the majority of the miR159 accumulated in HMW fractions previously identified as AGO1 associated HMW-RISCs (Fig. 1B). However, miR159 species exhibiting correct size were also detectable in fractions representing LMW-RISC and free sRNAs in pericarp samples. RNA content of the pooled FPLC fractions (Fig. 1B), and input samples of the various tissues were collected and subjected to high-throughput small RNA sequencing (HTS) library preparation using TruSeq Small RNA Library Preparation Kit (Illumina, San Diego, CA, USA) with the modified protocol described earlier (Czotter et al. 2018).

**Fig. 2:**
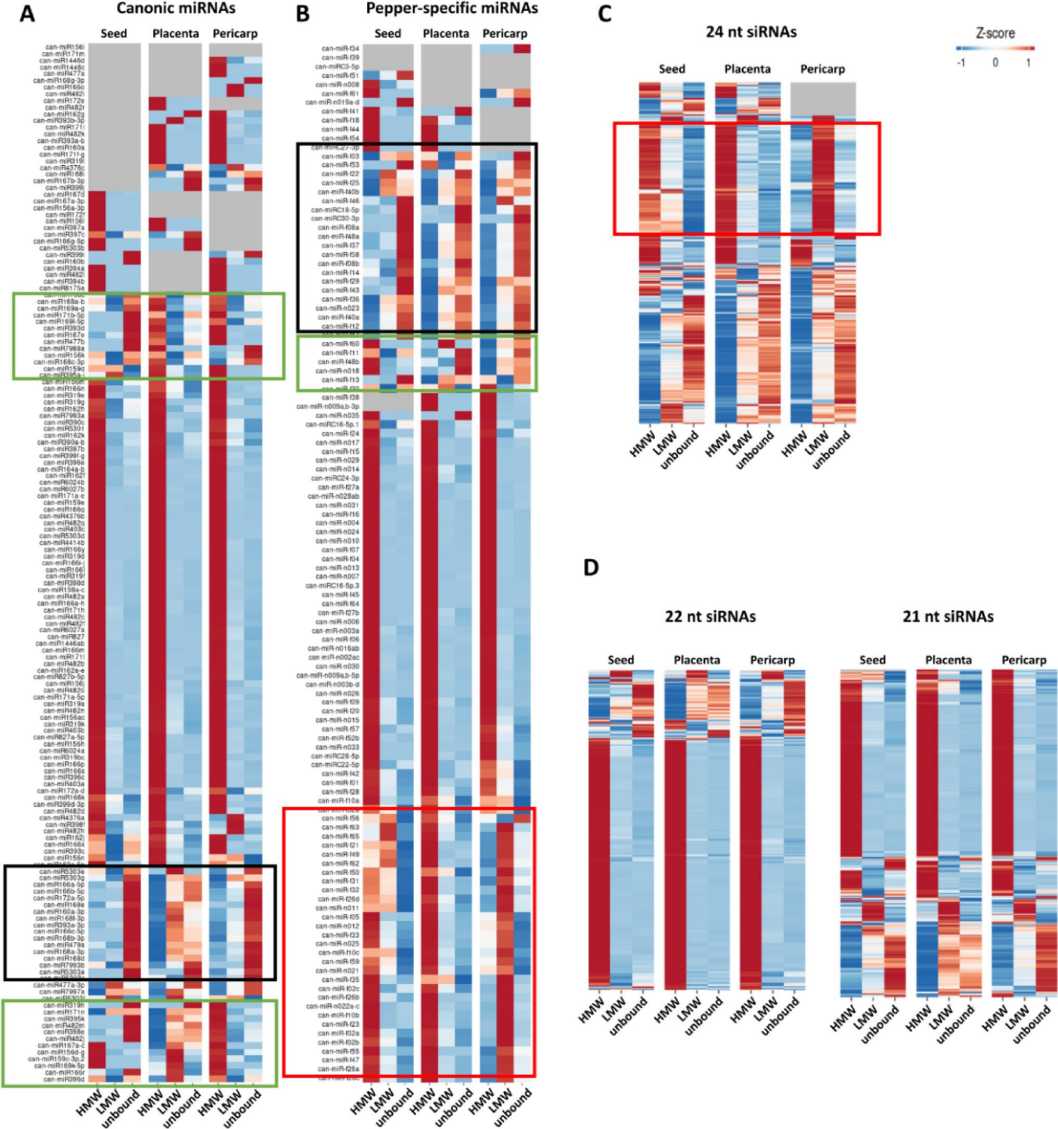
Z-score based heat map of canonical miRNAs **A**, predicted pepper specific miRNAs **B**, and 24-22-21 nt long siRNAs **C**, **D**. MiRNAs with variable distribution pattern are highlighted with green frames, black labels the poorly loaded miRNAs in at least one tissue. SRNAs in the red frame show a high abundance in seed and placenta HMW-RISC and pericarp LMW-RISC

**Fig. 3:**
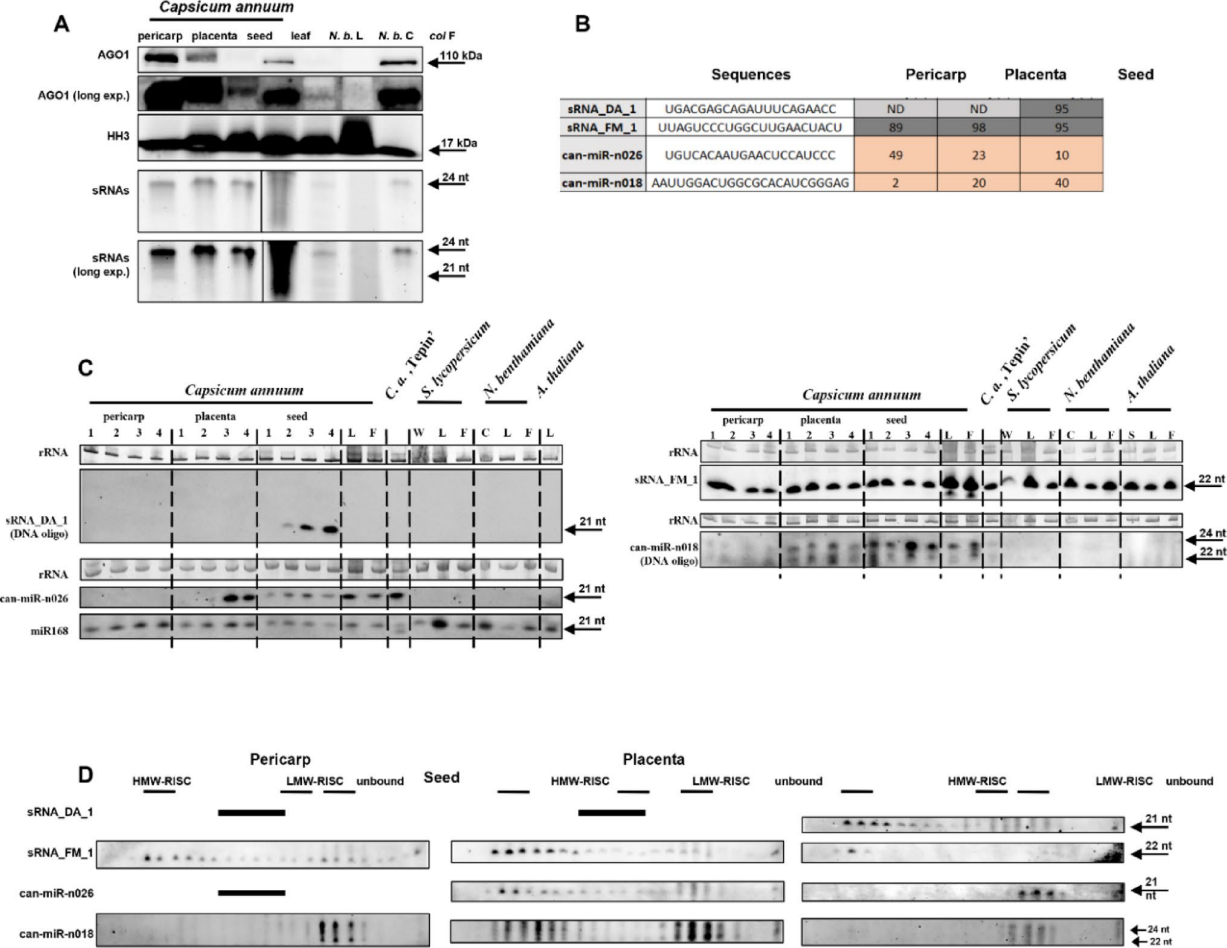
**A** AGO1 protein abundance and small RNA prevalence in pepper fruit tissues. In western blot Histone H3 serves as loading control. Small RNAs were stained with SYBR Gold. **B** Loading efficiency of selected small RNAs calculated based on the HTS data. **C** Expression analysis of the selected small RNAs in Capsicum annuum fruit pericarp, placenta, seed, leaf (L) and flower (F), Capsicum annuum var. aviculare ‚Tepin’ whole fruit, Solanum lycopersicum whole fruit (W), leaf (L), flower (F), Nicotiana benthamiana capsule (C), leaf (L) and flower (F), Arabidopsis thaliana leaf (L), silique (S) and flower (F). Numbers label samples of 14, 21, 28 and 40 DPA fruits, respectively. MiR168 is a positive control of the small RNAs. sRNA_DA_1 and can-miR-n018 were hybridized using DNA probes, sRNA_FM_1 and can-miR-n026 with LNA probes. **D** Distribution patterns of the selected small RNAs in gel-filtration experiments. Black arrows indicate the length of sRNAs

Two biological replicates of independent gel-filtration experiments from three tissue types were used for sRNA HTS representing input, HMW-RISC, LMW-RISC and AGO-unbound (free) samples originated from 28 days old pepper fruit dissected into pericarp (flesh), placenta and seed. As a quality check, the sequencing data of the prepared libraries were trimmed, filtered for tRNA or rRNA associated reads, and the 20–25 nt long sequences were mapped to the genome. The read numbers of the libraries varied between 1.5 and 42 million (Suppl. Figure 2 A). According to the principal component analysis, most of the variance of abundances between samples can be explained with the different gel-filtration pools (approximately 40%), while the different tissues are responsible only for about 18% (Suppl. Figure 2B). Notably, biological replicates clustered together. As for the tissues, seed is more distinguished, while placenta and pericarp (flesh) show higher similarity to each other regardless of the pool. HMW-RISC samples separate more from the unbound sRNA samples than the LMW-RISC samples. As expected from previous works, the size distribution of all reads of input samples shows the dominance of the 24-nt-long small RNAs, representing mostly the probably AGO4-associated siRNAs (Fig. 1C). Due to technical challenges only two replica experiments were carried out but the size distribution patterns of the two biological replica experiments show a strong correlation indicating the reliability of the results (Fig. 2C and D). In line with this, SYBR Gold staining also showed the predominance of 24-nt-long small RNAs over the 21-nt ones (Fig. 3A).

To date, only HTS analyses of gel-filtered sRNA pools of the model plant *Arabidopsis* leaves and flowers are available (Dalmadi et al. 2019). Similarly, we show that gel-filtration analyses can reveal distinct distribution patterns of the various sRNAs in the different pepper tissues. These data confirm that size dependent sub-arrangement of sRNA pools detected in *Arabidopsis* are also valid for pepper tissues. However, regarding sRNA size distribution there are clear differences between the various tissues.

In line with previous data, the 21-nt and 22-nt long small RNAs typically accumulated in a relatively higher proportion in HMW-RISC compared to the input, especially in the case of fruit pericarp (Fig. 1C). This increase of 21-nt and 22-nt sRNA species, typically miRNA-like sequences, in HMW-RISC was especially pronounced when sRNAs with RPM higher than 5 were investigated (Fig. 1D). We found that the dynamically expanding pericarp accommodated the highest level of miRNA-like sequences indicating the importance of miRNA mediated control of cell expansion and divisions. The portion of 21-nt and 22-nt long sRNAs also increased, although with a lesser extent, in HMW-RISC of placenta and seed samples relative to the 24-nt species. In addition, this effect was more pronounced in case of sRNAs with higher read numbers indicating that 24-nt sRNAs associated with HMW-RISC exhibit low abundances. In pericarp, the majority of the 24-nt and 23-nt long sRNAs with low read numbers, typically genome-associated siRNAs, accumulated in LMW-RISC and unbound sRNA pools similarly to the *Arabidopsis* model system (Dalmadi et al. 2019). However, this distribution was less pronounced in the case of sRNAs possessing RPM higher than 5. In contrast to *Arabidopsis* leaf and flower samples, pepper placenta and seed samples showed remarkably high level of 24-nt and 23-nt long sRNAs in HMW-RISC even in case of more abundant (higher mean value than 5 RPM) sRNAs besides to their canonical presence in LMW-RISC and unbound pools (Fig. 3C and D). These data indicate that a 24-nt mediated RNAi pathway might have an important control function through HMW-RISCs during fruit development. The presence of 24-nt siRNAs in RISC-associated samples is especially dominant in seeds which could be connected to the development of endosperm and reproductive organs (Grover et al. 2020). The abundant pools of AGO-unbound (free) sRNAs in all sizes were detected in every sample. This finding supports further our previous observations that in the cells a biologically relevant subset of sRNAs is present in protein unbound form. The potential functionality of this class of sRNAs remains to be elucidated in the future.

### HTS analysis reveals various loading efficiencies of the sRNAs in pepper fruit tissues

Next, mature sequences of canonical miRNAs (obtained from miRBase), and previously identified pepper-specific miRNAs were collected from the sequenced dataset and analyzed for their distribution between the fractions separately. On the heat maps z-scores of individual sRNAs are displayed. This shows the extent of deviation from the mean read number of the given sRNA in all samples generating relative values representing the distribution of the sRNA amongst the pools, but not the absolute abundance of the sRNAs. Current calculations were based on the read numbers of the sequenced pools (Suppl. Table 1). We observed highly variable distribution patterns of the individual miRNAs in both groups. In agreement with our previous work on *Arabidopsis* (Dalmadi et al. 2019), we were able to render the miRNAs into tissue-specific, highly HMW-RISC loaded, variably loaded and less active clusters based on their loading efficiency. Numerous miRNAs were associated with the HMW-RISC in every tissue (Fig. 2A and B). A major group of the variably loaded canonical miRNAs show a higher loading efficiency in pericarp (green frames on Fig. 2A). This indicates a presumed higher AGO1 content, as based on our previous observation, the loading efficiency of a miRNA depends on the AGO1 concentration (Dalmadi et al. 2019).

This presumption was verified by western blot presenting the highest AGO1 level in pericarp, a modest level in placenta and the lowest, but still detectable, level in seeds (Fig. 3A). Amongst the miRNAs previously identified as canonic or pepper specific, there are subgroups showing low or insignificant loading in certain tissues indicating reduced or no functionality at this developmental stage despite their notable abundance (black frames on Fig. 2A and B). Intriguingly, in case of pepper specific miRNAs, a subgroup of the sequenced reads associates with LMW-RISC in pericarp, while in the rest of the tissues they occur prevalently in HMW-RISC (red frame on Fig. 2B). The loading efficiency showed no correlation with the abundance of the sRNAs (Suppl. Figure 3) as it was expected from previous studies. In line with size distribution data analyses, we observed abundant presence of 24-nt siRNA species associated with the HMW-RISC in seed and placenta samples. However, we also observed a drastic shift of these siRNA species to LMW-RISC in pericarp (red frame Fig. 2C). These latter findings may suggest the presence of altered type of RNAi pathways in the growing pepper fruit. Since 24-nt siRNAs are predominantly associated with RNA-dependent DNA methylation (RdDM) pathway this finding may point to the specific action of RdDM in pericarp. In addition, other siRNA species, like 21-nt and 22-nt long siRNAs, are in a surprisingly high number represented in the HMW-RISC in all tissue types showing high loading efficiency (Fig. 2D). Altogether, similarly to other plant species, we can form clusters based on the distribution pattern of different miRNAs in different tissues as highly, low and variable loaded miRNAs. Consequently, there are miRNAs with the same distribution pattern in every tissue type but also there are miRNAs behaving differently in various tissues.

### Identification of potentially biologically active sRNAs based on AGO-loading capacity

To identify active, highly AGO-bound sRNAs, HTS data were analyzed, and loading efficiency was calculated for the sequences having mean read count higher than 50 RPM. Those having in at least one tissue higher loading efficiency than 80%, were selected. As another strategy, from the sequences filtered for higher abundance those were selected which had a loading efficiency lower than 50%. From these groups two representative sRNAs, having only one hit on the genome, were selected to be tested for their species and organ specificity, expression patterns during the development and distribution amongst the gel-filtration fractions. sRNA_DA_1 was selected as a highly loaded tissue specific sRNA, having a single target, a predicted receptor-like kinase RBK2 (NC029984.1). Based on the sequencing data this 21 nt-long sRNA starting with uracil is expressed in seeds and 95% of it is associated with the HMW-RISC pool (Fig. 3B). Northern blot using DNA probe detected solely the 21 nt long sRNA in pepper seed of fruits 21, 28 and 40 DPA, and not in other species (Fig. 3C). Using LNA modified version of the same probe we were able to detect the 21-nt version of this sRNA in pepper and *N. benthamiana*, and an approximately 24-nt long un-specific signal in every sample, as well (Suppl. Figure 4). HTS data confirmed that no 24-nt version of sRNA_DA_1 has been found (Suppl. Figure 5) so this signal can be considered as unspecific. The finding that 21-nt signals were not detected by DNA probe in other species indicates either the absence or the low expression level of sRNA_DA_1 in these immature whole fruit samples. Gel-filtration of 28 DPA seed sample confirmed the good loading ability of the 21-nt sRNA_DA_1 into the HMW-RISC (Fig. 3D). The strong spatiotemporal expression pattern and the high loading efficiency indicates potential functionality of this previously unknown sRNA in the seeds of the developing pepper fruit.

As another example of the newly identified sRNAs, sRNA_FM_1 was identified based on its abundance and high calculated loading efficiency in the HTS data (Fig. 3B). It corresponds to a single locus on the genome. Its presumed target is a Squamosa promoter-binding-like (not miR156 target) protein (LOC107871232). This previously non-characterized sRNA is abundantly expressed in all species, tissues and developmental stages investigated in the current study (Fig. 3C). Although no known function was previously described for this yet unidentified sRNA, the high loading efficiency observed in all pepper fruit tissues suggests a role in fruit development (Fig. 3D).

Our experimental setup also allowed us to investigate the expression pattern and loading efficiency of predicted miRNAs formerly identified as pepper specific. As an example, can-miR-n026 was selected as presenting a variable distribution pattern in HTS data. This miRNA induces phasiRNA biogenesis by cleaving the transcript of receptor-like protein genes. Taking its specificity into account, can-miR-n026 is indeed pepper specific and detected with northern blot in 28 DPA and 40 DPA placenta, in seed, leaf, and flower (Fig. 3C). It was previously described, based on its high abundance, as a miRNA having a major role in fruit development. In contrast, in fruit pericarp it is not expressed at all and a northern blot of gel-filtration fractions revealed that the majority of the miRNA exists in the inactive AGO-unbound pool (free) in seeds. Higher loading efficiency was solely detected in 28 DPA placenta suggesting a special, localized functionality (Fig. 3D).

Finally, can-miR-n018 was investigated as a reportedly pepper-specific miRNA with high abundance in HTS data. Both LNA and DNA based probes detected hybridization signal pattern corresponding to sRNAs 22-23-24-nt in length (Fig. 3C and Suppl. Figure 4). The various can-miR-n018 versions appear to be iso-MIRs, and based on the HTS data, only a subfraction of the reads correspond to the previously described pepper specific 24 nt-long miRNA. None of the iso-MIRs shows high AGO loading efficiency neither in HTS data nor gel-filtration experiments in pericarp and seed but all iso-MIRs loaded in HMW-RISC with moderate efficiency in placenta (Fig. 3D).

## Conclusion

Following the discovery of RNAi pathways, a worldwide research effort was evoked to identify new siRNAs and miRNAs of scientific and/or agronomic importance in the hope of understanding major molecular events behind certain biological processes. During these efforts the expression of individual sRNAs or whole sRNA content of the investigated tissue was determined, and the conclusion about functionality was drawn solely by the presence and abundance of certain sRNAs. Lately, an abundant AGO-unbound sRNA pool was discovered in plant cells suggesting that only a sub-population of the sRNAs is in active AGO-bound form (Dalmadi et al. 2019). The distribution of sRNAs between the AGO-RISC loaded and unbound forms is characteristic of the individual sRNAs. In this way, the potential functionality can be determined by the combined investigation of abundance and loading efficiency, and only those sRNAs can be considered important in given tissues, which are not just present, but exist at least partly in an AGO-bound form. The method presented here can also highlight such unknown sRNAs, which were otherwise not discovered, but can be active in the investigated tissue, and consequently should have a regulatory role. The FPLC-based gel-filtration allows the simultaneous observation of all RISC-associated sRNAs, regardless of the AGO content of the complex, and the AGO-unbound pool from the same tissue.

In this work we have demonstrated that the phenomenon of tissue specific sorting of sRNAs into AGO proteins containing HMW- and LMW-RISCs as well as protein unbound pools is also present in economically important pepper plant. From practical point of view, we propose that gel-filtration based size separation of sRNA complexes can be a useful tool to identify potentially biologically active sRNA species at tissue specific manner based on their ability to be loaded into the executor AGO proteins. The identification of candidate sRNAs via this technology can open the way to experimental validation of the function of these potentially active sRNAs.

Based on bioinformatic analysis, 12 *AGO* genes were identified from pepper, from which two seemed to be pseudo-genes (Qin et al. 2018), but there is only AGO1 antibody available at the moment. In the lack of other AGO antibodies future experiments will be needed to assess the precise AGO content and (Qin et al. 2018) AGO distribution between HMW- and LMW-RISCs of various tissue samples. In case of availability, pepper specific AGO antibodies used in western blot and immune-precipitation experiments will be helpful to reveal the association of selected sRNAs and specific AGO proteins in HMW- and LMW-RISC pools. One of our findings in the pepper system is the presence of AGO-unbound sRNA pool in all tissue context similarly to other plant systems. This observation again raises the question of the functionality of this pool whether these sRNAs are superfluous biogenesis products deposited in the cytoplasm or could be a reservoir for rapid AGO loading responding to stress factors or developmental cues. The investigation of this phenomenon requires elaborated technological tools, such as in vitro systems, transgenic plants, transient expression systems, specific antibodies which are not available in the case of pepper. We envision that other more elaborated model system, like *Arabidopsis thaliana*, will be more suitable for this purpose in future experiments. As these technological shortages hinder the fast functional analyses of sRNAs in pepper system, gel-filtration based analysis of the RISC containing and AGO-unbound content of the given tissue type could be a resource of information about the functionality of sRNAs. The presence of 24-nt siRNAs in HMW-RISC is interesting, and it suggests the formation of a yet unknown RISC, which maybe contains AGO species other than AGO1. Further experiments to determine the AGO-content of RISCs in these tissues, and the association of sRNAs with these AGOs would be elaborative in terms of this issue. Lacking the suitable techniques in pepper, we could only presume that these 24-nt long sRNAs take part in the RdDM pathway.

It is important to note that HTS of the collected HMW-RISC, LMW-RISC and AGO-unbound pools has its drawbacks due to the different sequencing biases (Sorefan et al. 2012). Due to sequencing bias, there could be some distortion in case of certain sRNAs, since the sRNA composition of the various cloned pools differs and consequently the sequencing bias is not exactly the same comparing HMW-, LMW-RISC and unbound pools. This can cause some alterations in the calculation of AGO loading efficiency, but does not interfere with the clustering of highly and low loaded sRNAs. Due to this potential technological drawback during the functional investigations of selected sRNA it is recommended to validate the HTS results using laboratory experiments such as sRNA northern blots or qPCR analyses.

In spite of this technological aspect, this method can serve as a valuable resource of data taking RISC loading efficiency into account which is inevitable when searching for potentially functional sRNAs. Our work draws attention to the notion that the RISC loading efficiency as an indicator of biological activity has to be also considered together with the changes in abundance to successfully identify active sRNAs.

## Supplementary Information

Below is the link to the electronic supplementary material.

Supplementary Material 1

Supplementary Material 2

## Data Availability

Raw data from the RNA have been deposited in the NCBI BioProject database under the ID PRJNA1090017.
