# Supplementary material for: Tissue specific RISC-loading reassesses small RNA functionality in developing pepper fruit
Source: Plant Mol Biol. 2026 Jul 11;116(4):69. doi: 10.1007/s11103-026-01742-6 (PMC13356050; doi:10.1007/s11103-026-01742-6)
Supplement: Supplementary file 1 — Supplementary Material 1 [file 11103_2026_1742_MOESM1_ESM.pdf]

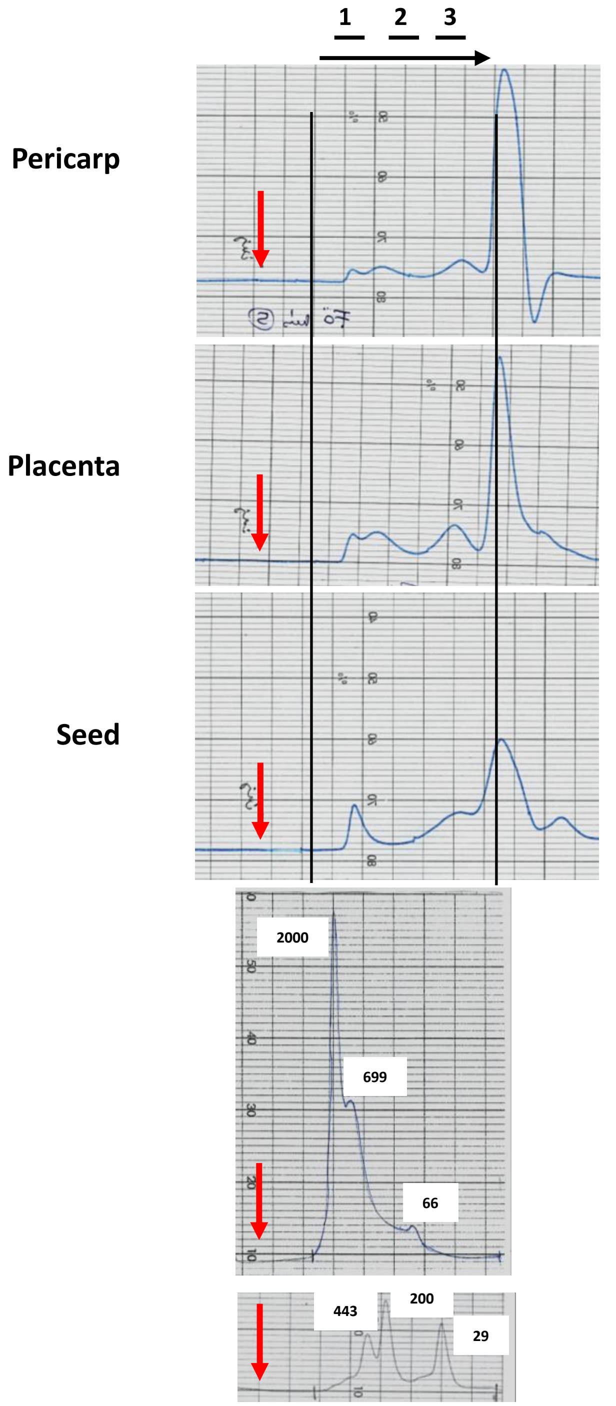

Supplementary Figure 1: FPLC conditions presented with the chromatograms of representative runs of pericarp, placenta and seed samples. All the runs were prepared subsequently on the same coloumn, using the same parameters. Fractions were collected within the time frame indicated by the vertical black lines. Black arrow shows the direction of the run, red arrow indicates the timepoint of injections. Positions of HMW-RISC (1), LMW-RISC (2) and unbound (3) fractions on runs of this coloumn were experimentally defined from several species before (*Arabidopsis th., Nicotiana b., Hordeum v.*) alongside protein size markers (Carbonic Anhydrase, 29 kDa; Albumin, 66 kDa; Alfa-amylase, 200 kDa; Apoferritin, 443 kDa; Thyroglobulin, 699 kDa; Blue dextran, 2000 kDa).

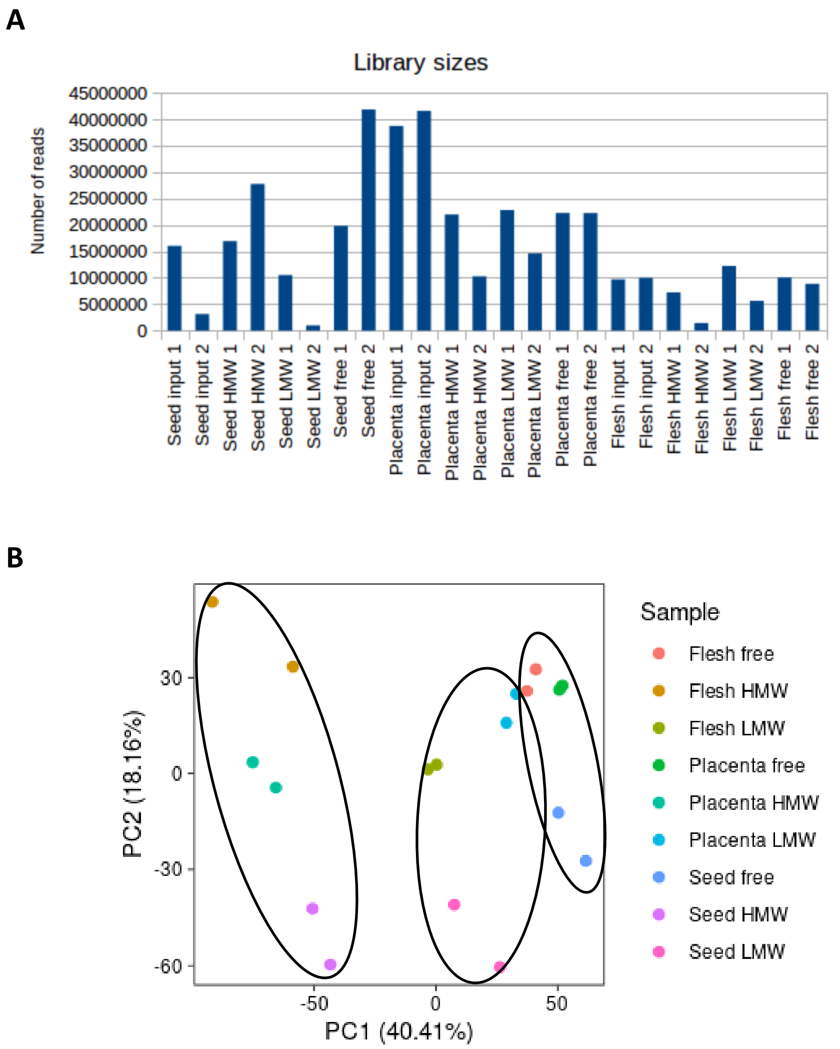

Supplementary Figure 2: A Read numbers of the sequencing libraries. B Principal Component Analysis of the HTS data. Samples of individual pools were circled together.

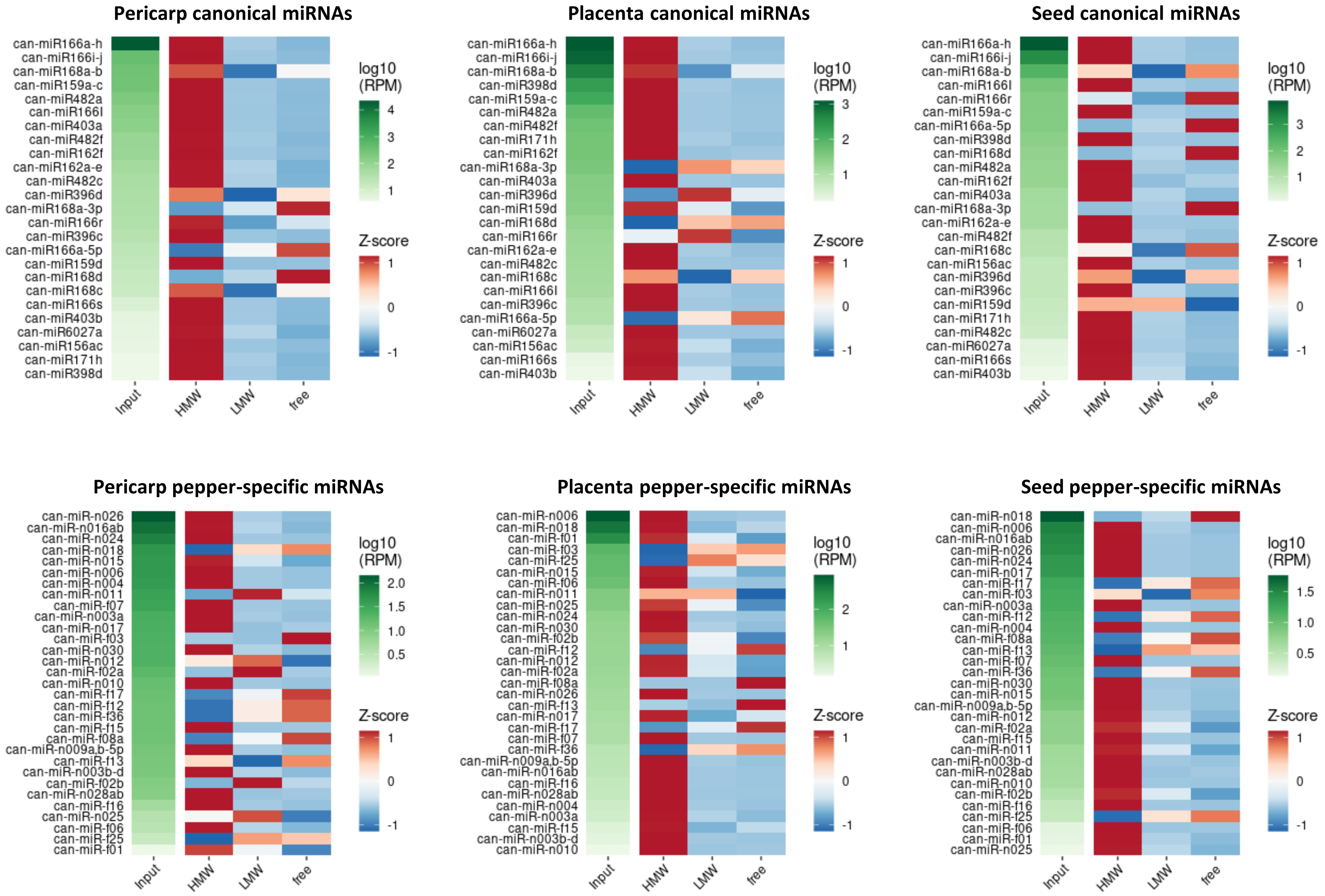

Supplementary Figure 3: Z-score based heat map of canonical and pepper-specific miRNAs ordered according to the total abundance in input samples.

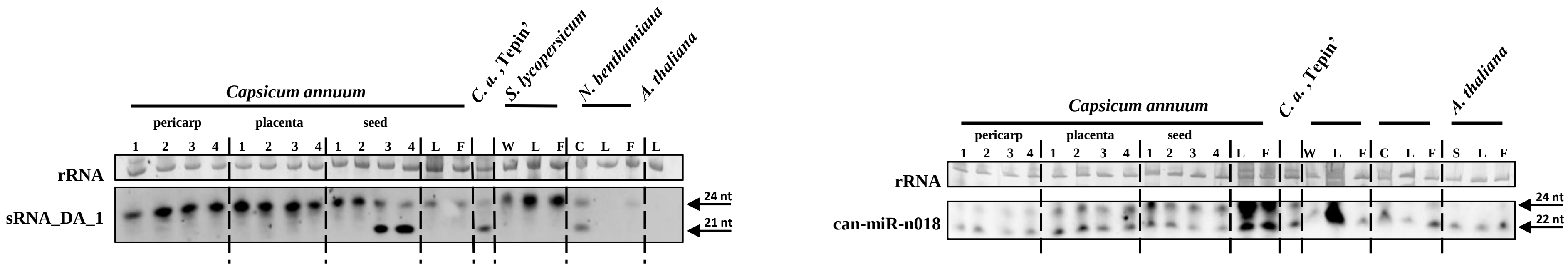

Supplementary Figure 4: Expression analysis of sRNA_DA_1 and can-miR-n018 using LNA probes in *Capsicum annuum* fruit pericarp, placenta, seed, leaf (L) and flower (F), *Capsicum annuum var. aviculare* ,Tepin' whole fruit, *Solanum lycopersicum* whole fruit (W), leaf (L), flower (F), *Nicotiana benthamiana* capsule (C), leaf (L) and flower (F), *Arabidopsis thaliana* leaf (L), silique (S) and flower (F). Numbers label samples of 14, 21, 28 and 40 DPA fruits, respectively. Black arrows indicate the length of sRNAs.

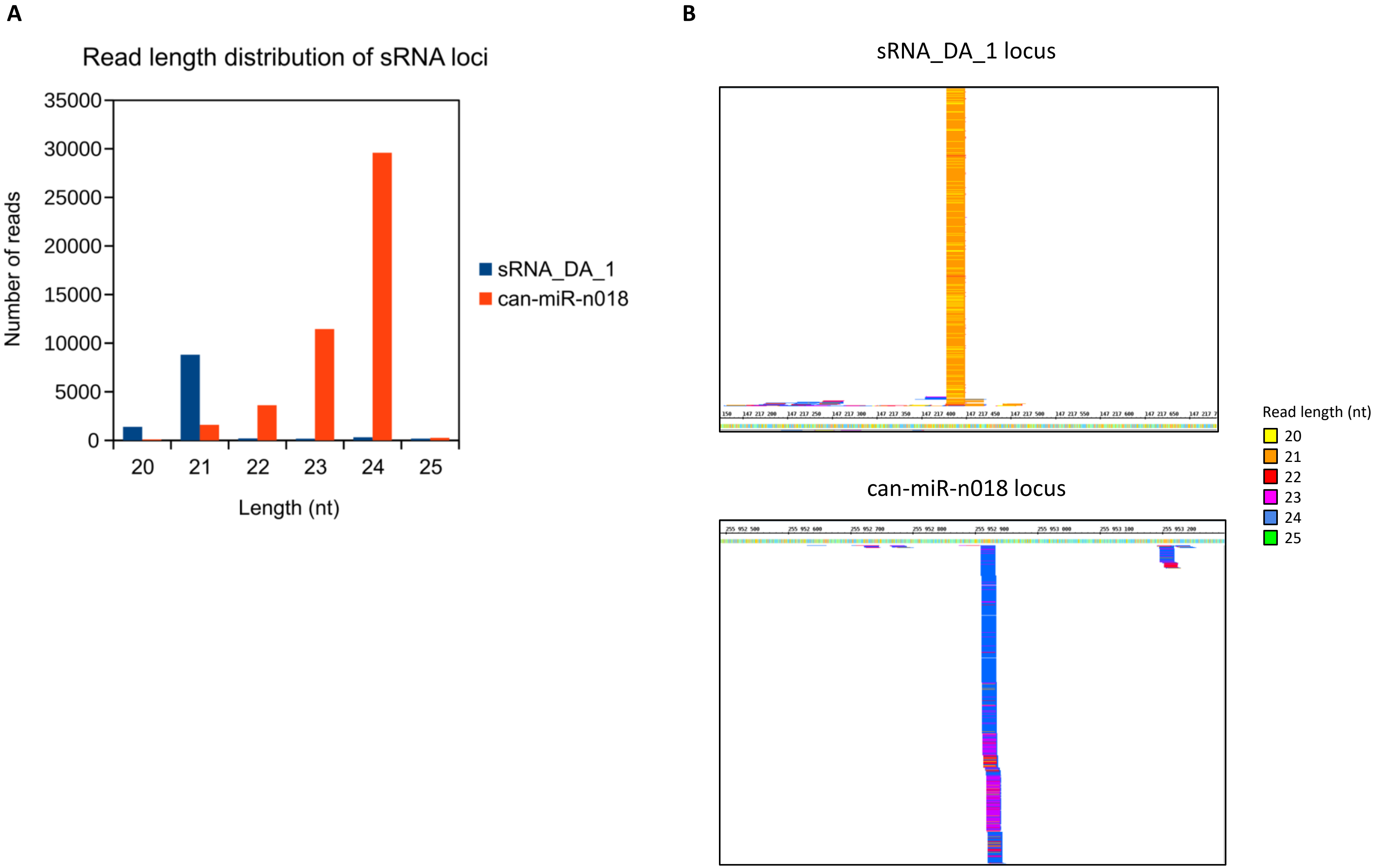

Supplementary figure 5: A Read length distribution along the sRNA loci. Reads of the input samples of all tissues were merged and reads mapped to the sRNA loci were counted. **B** Genome browser view of the loci showing the alignment of the reads colored by length. The sRNA_DA_1 locus is dominated by the major 21-nt-long sRNA, while the can-miR-n018 is dominated by the major 24-nt-long sequence and its 23-nt-long isoform. Both loci are stranded, no phased siRNAs are produced from them.
